# Forage Plant Ecophysiology under Different Stress Conditions

**DOI:** 10.3390/plants13101302

**Published:** 2024-05-09

**Authors:** Agustín A. Grimoldi, Carla E. Di Bella

**Affiliations:** IFEVA, Universidad de Buenos Aires, CONICET, Facultad de Agronomía, Buenos Aires C1417DSE, Argentina

## 1. Introduction

Forage production often occurs in fragile environments with low fertility and various limitations. The main topic of this Special Issue is the study of the effects and new mechanisms of tolerance and recovery under different environmental stress conditions in forage species. Furthermore, climate change could increase the likelihood of several stressful events, such as heavy rainfall leading to soil waterlogging or submersion, extreme temperatures, and drought conditions, negatively impacting plant growth and productivity [1,2]. New livestock production systems are also common under trees or shrub cover, where forage plants grow in varying degrees of shade. In turn, each of these abiotic stresses generally acts in combination with defoliation or with another of the stressors (e.g., flooding and salinity, drought, and heat stress, among others). They can even act in different temporal sequences in relation to the environmental variability of the system, which was also increased by climate change. In general, information is available on the response to each individual stress, but less is known about the ecophysiological mechanisms involved in the tolerance to the combination and temporal sequences of different types of stress. Understanding the effects and mechanisms of tolerance and recovery under abiotic stress conditions is crucial as a foundation for the genetic improvement of forage species and to develop optimal grazing management strategies that promote the production, quality, and persistence of valuable species and environmental sustainability.

In this context, the aim of this Special Issue is to enhance our understanding of novel mechanisms of tolerance to stresses and their patterns of variation within and between accessions of different forage species. We present six scientific articles authored by individuals affiliated with various countries, including Argentina, Spain, Ethiopia, Brazil, Kenya, Australia, and Fiji. This results in the analysis of different stresses specific to various pastoral systems worldwide. Additionally, studies on grasses and legumes were conducted. These works underscore the significance of studying genetic variability as a crucial initial step in identifying tolerant accessions and signify clear progress in elucidating mechanisms of tolerance. However, we are convinced that this topic still warrants attention from the scientific community.

## 2. Advances in Forage Plant Ecophysiology under Different Stress Conditions

In the following paragraphs, we present a summary of the main findings reported by different research groups in this Special Issue.

Negawo et al. [3] analyzed the genetic diversity of the *Sesbania sesban* (i.e., a forage legume tree) collection using genome-wide markers, revealing higher variability within accessions than between them. Besides, a lack of relationship between the genetic variation of the germplasm and its geographical origin was found. A representative subset of 34 accessions with diverse origins and agro-ecologies was developed using SNP markers, providing valuable information for future improvement programs to develop high-yielding, stress-tolerant varieties for crop-livestock-based production systems.

Habte et al. [4] evaluated 84 Napier grass (i.e., *Cenchrus purpureus*, perennial tropical forage grass) genotypes for drought stress tolerance. Results showed genotype variation; the growth and productivity of the genotypes declined under severe water stress conditions compared to moderate stress conditions. High biomass-yielding genotypes with enhanced WUE were consistently observed across harvests in each soil moisture stress regime. In addition, the top biomass-yielding genotypes produced the highest annual crude protein yield, suggesting the potential for developing high-feed-quality Napier grass cultivars suitable for drought-prone environments.

Schulz et al. [5] aimed to improve pasture utilization efficiency by understanding leaf generation dynamics and their responses to nitrogen fertilization in *Paspalum notatum* (i.e., forage tropical grass) genotypes. The study found that increased biomass production after nitrogen fertilization was due to higher tiller density and tiller weight, with tiller weight influenced by leaf blade length and width. Seasonal variation in biomass production was mainly explained by changes in leaf blade length, and different genotypes showed varying morphogenetic traits, suggesting the need for different management practices.

Marinoni et al. [6] evaluated plant functional traits in six populations of false Rhodes grass species (i.e., *Leptochloa crinita* and *L. pluriflora*; native forage grasses for arid and semiarid rangelands in Argentina and the United States) from different regions. It found a fixed ontogenetic variation in seed weight across environments, and seed weight played a crucial role in germination under osmotic stress. The research highlighted the significance of seed weight in seedling survival under arid conditions, while other traits were important in later growth stages.

Borrajo et al. [7] focused on tall wheatgrass (i.e., *Thinopyrum ponticum*; temperate forage grass) and its response to the combination of drought and salinity conditions. Moderate drought or salinity stress resulted in higher water-use efficiency, proline levels, and certain leaf traits compared to control conditions. Different accessions showed distinct strategies in response to stress, with some prioritizing reproductive development (those from environments with mild/moderate stress) and others emphasizing vegetative development (those from environments with strong drought and salinity). The study suggests that specific traits, such as 13C value, Na+/K+ ratio, and canopy structural variables, can be used to identify well-adapted accessions for forage production under changing climate conditions.

Mollard et al. [8] investigated the effects of sequential water stress (drought followed by waterlogging or vice versa) on *Chloris gayana* (i.e., tropical forage grass). The research found that while both waterlogging and drought reduced plant growth rate similarly in the first round of stress, the plants’ high recovery ability outweighed any acclimation to the previous stress when facing a second round. The study suggests that the grass’s tolerance to sequential water stress depends more on its recovery ability than on previous exposure, which could be valuable in breeding forage grasses for poorly drained soils experiencing sequential stress events.

## 3. Future Perspectives

The climate in the world is gradually changing, and therefore agricultural activities will face new challenges in the future to sustain their production. Climate change will lead to the occurrence of increasingly dynamic environmental scenarios [9]. Therefore, the challenge for researchers in the ecophysiology of forage plants will be to generate scientific advances that increase resilience, that is, the ability of plants to recover from stress and improve their persistence in each type of environment. For this, the following two fundamental aspects must be considered as future perspectives: (i) the underlying mechanisms and their variability within and between accessions of the responses to combined stress and the occurrence of different temporal sequences of stress, and (ii) in all cases, the need to evaluate after the post-stress recovery period.

Although plants deal with combined and sequential stresses in nature, so far, most studies in agricultural crops address adaptive responses to single stress events. A relatively new and increasingly common situation is that an intense stress event can be followed by conditions of another type of stress. Therefore, it is important that perennial forage species have tolerance to stress sequences characteristic of the target environment of their implementation, thus improving their persistence and optimizing the sustainability of the production system. Then, it is necessary to deepen the knowledge of whether the tolerance responses to each type of stress are divergent or convergent and eventually produce cultivars better adapted to the most probable sequence of stresses in each of the environments.

Furthermore, stress recovery, and thus effective plant persistence, is rarely reported [10,11,12,13]. It is our knowledge that the resumption of plant growth after a period of stress is essential to evaluating their tolerance. In certain cases, the tolerance of different accessions to a certain environmental condition is not evident during the stress period, either because the plants do not show growth (performing a quiescence strategy) or grow little during the stress; and the real differences in tolerance only become evident once they resume growth. The mechanisms involved in the recovery of growth rates after a stress condition are still poorly understood.

The demand for forage in situations of abiotic stress, and even more so the greater frequency of intense climatic oscillations, makes it necessary to increase stress tolerance in forage species and thus sustain the productivity, persistence, and sustainability of livestock systems in these new productive scenarios. Thus, the first step is to evaluate the genetic variability and heritability of the characters involved. For the continuation of these lines of research, the priority objective is to make available to the productive sector, in an explicit and transferable manner, novel information based on the identification of heritable ecophysiological characters associated with tolerance to different environmental conditions (flood, drought, heat stress, salinity, nutrient deficit, soil acidity, and toxicity), and in particular in their possible combinations and stress sequences given the greater climate variability expected for the coming decades.

## Data Availability

Data sharing is not applicable.
